# Postural changes in women with chronic pelvic pain: a case control study

**DOI:** 10.1186/1471-2474-10-82

**Published:** 2009-07-07

**Authors:** Mary LLS Montenegro, Elaine CL Mateus-Vasconcelos, Júlio C Rosa e Silva, Francisco J Candido dos Reis, Antonio A Nogueira, Omero B Poli-Neto

**Affiliations:** 1 Department of Gynecology and Obstetrics, Faculty of Medicine of Ribeirão Preto, University of Sao Paulo- Ribeirao Preto, São Paulo, Brazil; 2 Department of Surgery and Anatomy, Faculty of Medicine of Ribeirão Preto, University of Sao Paulo- Ribeirão Preto, São Paulo, Brazil

## Abstract

**Background:**

Chronic pelvic pain (CPP) is a lower abdominal pain lasting at least 6 months, occurring continuously or intermittently and not associated exclusively with menstruation or intercourse. Although the musculoskeletal system has been found to be involved in CPP, few studies have assessed the contribution of posture in women with CPP. We aimed to determine if the frequency of postural changes was higher in women with CPP than healthy subjects.

**Methods:**

A case-control study included 108 women with CPP of more than 6 months' duration (CPP group) who consecutively attended at the Hospital of the University of São Paulo and 48 healthy female volunteers (control group). Postural assessment was noninvasive and performed in the standing position, with the reference points of Kendall used as normal parameters. Factors associated with CPP were assessed by logistic regression analysis.

**Results:**

Logistic regression showed that the independent factors associated with CPP were postural changes in the cervical spine (OR 4.1; 95% CI 1.6–10.7; p < 0.01) and scapulae (OR 2.9; 95% CI 1.1–7.6; p < 0.05).

**Conclusion:**

Musculoskeletal changes were associated with CPP in 34% of women. These findings suggest that a more detailed assessment of women with CPP is necessary for better diagnosis and for more effective treatment.

## Background

Among women, chronic pelvic pain (CPP) is a highly prevalent (2% to 25%) clinical problem [1,2], with substantial costs [3] as well as social and marital repercussions [4,5]. CPP is defined as continuous or recurrent pain in the lower abdomen or pelvis lasting at least six months, not related to pregnancy, and sufficiently severe to interfere with the habitual activities of the patient. CPP excludes pain occurring exclusively in association with menstruation (dysmenorrhea) or during sexual intercourse (dyspareunia).

Although the etiology is often unknown, it may result from complex interactions among the gastrointestinal, urinary, gynecologic, musculoskeletal, neurologic and endocrine systems, as well as being influenced by psychological and sociocultural factors [6]. To date, few therapeutic modalities have been effective in relieving the symptoms of CPP, particularly over the long term [7]. An interdisciplinary approach has therefore been recommended [8-10], both to diagnose the presumed primary etiology, and to diagnose and control all the secondary factors associated with CPP.

In clinical practice, postural changes are frequently observed among women with CPP. Although this disease has been associated with musculoskeletal changes [9] and particular postures [11], to date there have been no studies of the detailed postural evaluation of women with CPP, which can be performed by attending physicians, especially primary care physicians [5] and gynecologists [12]. Postural assessment can lead to early detection of uneven positions, shortenings, antalgic postures and tensions. Although these changes may not be the primary cause of the clinical condition, they can contribute significantly to the worsening of pain and tension. We therefore determined the frequency of postural changes in women with CPP, as assessed only by clinical examinations.

## Methods

A case-control study was performed on 108 consecutive women with CPP of more than 6 months' duration (CPP group) examined at the Hospital of the University of Sao Paulo and on 48 healthy female volunteers (control group). The study was approved by our Research Ethics Committee and all participants gave written informed consent.

Before physical examination, each CPP patient filled out a detailed form containing information about the characteristics of her pain and her personal history, as well as completing the Beck Depression Index (BDI), Visual Analogue Scale (VAS) and the McGill Pain Index (MPI). The control group completed only the BDI (Table 1). Each patient and control subject underwent a physical examination consisting of a general evaluation, superficial and deep palpation of the abdominal wall, including Carnet test and investigation of trigger points. Postural assessment was noninvasive, carried out in standing position, with the reference points of Kendall [13] being used as normal parameters.

**Table 1:** Demographic and Clinical Characterization of the CPP and Control Groups.

	Control	CPP	p
Number of subjects	48	108	---
Age, years (mean ± SD)	31.8 ± 8.7	35.3 ± 8.6	ns
BMI, kg/m^2 ^(mean ± SD)	26.4 ± 4.7	26.0 ± 4.8	ns
No. of gestations (median, range) range)	2, 0–8	2, 0–6	ns
Abortion % (n)	14.6 (7/48)	20.7 (22/106)	ns
Episiotomy % (n)	29.2 (14/48)	35.2 (38/108)	ns
Cesarean section %, (n)	41.7 (20/48)	35.2 (38/108)	ns
Stable relationship % (n)	66.7 (32/48)	76.8 (83/108)	ns
Duration of symptoms (months) mean ±	---	60.9 ± 6.4	---
VAS (mean ± SD)	---	66.8 ± 2.1	---
McGill (mean ± SD)	---	28.7 ± 2	---
BDI (median, range)	8, 0–28	16, 3–42	<0.01
Dysmenorrhea % (n)	12.5 (6/48)	62 (67/108)	<0.01
Dyspareunia* % (n)	8.3 (4/48)	49.1 (53/108)	<0.01
Intestinal symptoms % (n)	4.2 (2/48)	41.7 (45/108)	<0.01
Urinary symptoms % (n)	12.5 (6/48)	47.2 (51/108)	<0.01

Notes- SD: standard deviation; BMI: body mass index; CPP: chronic pelvic pain; VAS: Visual Analogue Scale; BDI: Beck Depression Inventory.

*Considering only moderate or intense symptoms. If we also consider mild symptoms, control: 20.8% (10/48) × CPP: 75.9% (82/108).

Alterations analyzed on the sagittal plane included knee hyperextension; pelvic anteversion and retroversion; lumbar hyperlordosis and rectification; thoracic hyperkyphosis and rectification; protraction and medial rotation of the shoulder; cervical hyperlordosis and rectification; and head protraction. Alterations analyzed on the frontal plane included asymmetry of the malleoli; pes cavus and flat feet; valgum and varum knee; asymmetry of the iliac spine; lumbar scoliosis; head tilt and rotation; and winged, plane and discrepant scapulae. Tests to determine the difference in length of the lower limbs were performed to differentiate real and apparent discrepancies and fingertip to floor distance [14-16].

All subject evaluations were performed by two physical therapists who had worked together for four years and who were blind to all clinical data and clinical findings previously obtained by the physicians, including the condition of each patient or control subject.

### Statistical analysis

Cohen's kappa coefficient was used to determine inter-rater reliability. All discrepancies were re-examined until a consensus was reached by the examiners. Univariate and descriptive analyses were carried using the GraphPad Prism 4 for Windows software. Student's t test and the Mann-Whitney test were used to analyze continuous data with and without normal distribution, respectively, and the Chi-square test or Fisher's exact test was used to analyze categorical data, as appropriate. Logistic regression analysis was used to identify the independent variables significantly associated with CPP. For logistic regression analysis, we selected only those findings that were significant as determined by Fisher's exact or the Chi-square test, with values of 0 and 1 assigned to the absence and presence, respectively, of each variable in each subject. Logistic regression was performed using MedCalc Software Statistic (v9.4.0.2), with the level of significance set at p < 0.05.

## Results

We observed a postural difference between the CPP and control groups, although the difference was statistically significant (p < 0.01) only for the upper segments of the body, i.e., the cervical spine and scapulae. Observed postural changes and the indices of interobserver agreement are listed in Table 2.

**Table 2:** Postural changes in women with CPP and in healthy women.

Postural changes	Control (n = 48)	CPP (n = 108)	kappa	OR	95%CI	p
Head % (n)	72.9 (35)	84.3 (91)	0.53	1.2	0.5–2.9	NS
Tilt	72.9 (35)	83.3 (90)				
Rotation	---	46.3 (50)				
Protraction	6.2 (3)	27.8 (30)				
Cervical spine % (n)	12.5 (6)	36.1 (39)	0.85	3.9	1.5–10	<0.01
Rectification	6.2 (3)	6.5 (7)				
Hyperlordosis	6.2 (3)	29.6 (32)				
Shoulders % (n)	50 (24)	59.3 (64)	0.27	1.4	0.8–6.1	NS
Protraction	43.7 (21)	48.1 (52)				
Medial rotation	12.5 (6)	19.4 (21)				
Scapulae % (n)	12.5 (6)	27.8 (30)	1.00	2.8	1–7.5	<0.01
Winged	12.5 (6)	11.1 (12)				
Plane	---	4.6 (5)				
Discrepancy	---	13 (14)				
Thoracic spine % (n)	18.8 (9)	14.8 (16)	0.39	0.8	0.1–4.8	NS
Hyperkyphosis	12.5 (6)	1.8 (2)				
Rectification	6.2 (3)	13 (14)				
Lumbar spine % (n)	50 (24)	62 (67)	1.00	0.8	0.7–6	NS
Hyperlordosis	43.8 (21)	44.4 (48)				
Rectification	6.2 (3)	16.7 (18)				
Scoliosis	---	4.6 (5)				
Pelvis % (n)	62.5 (30)	76.8 (83)	0.54	2	0.3–3.2	NS
Antepulsion	25 (12)	32.4 (35)				
Asymmetry of the iliac spines	35.4 (17)	60.2 (65)				
Retroversion	2.1 (1)	1.8 (2)				
Knee % (n)	20.8 (10)	29.6 (32)	0.51	1.6	0.2–2.8	NS
Hyperextension	12.5 (6)	13 (14)				
Valgum	6.2 (3)	14.8 (16)				
Varum	2.1 (1)	3.7 (4)				
Feet % (n)	37.5 (18)	38 (41)	0.97	1	0.4–3.9	NS
Cavus	18.8 (9)	29.6 (32)				
Flat	8.3 (4)	6.5 (7)				
Asymmetry of the malleoli	10.4 (5)	2.8 (3)				
LDR (mean ± SD)	81.0 ± 10.2	83.4 ± 7.1				
LDA (mean ± SD)	88.8 ± 10.2	91.1 ± 7.3				
FFD (mean ± SD)	11.9 ± 9.7	13.9 ± 13.4				

Notes- LDR: Leg-length discrepancy real; LDA: Leg-length discrepancy apparent;

FFD: Fingertip to floor distance. Kappa: inter-rater reliability; SD: standard deviation; OR: odds ratio; CI: confidence interval; Ns: non-significant; CPP: chronic pelvic pain.

## Discussion

We observed statistically significant differences in the cervical spine and scapulae between women with CPP and control women. We believe that the changes observed in women with CPP resulted from a vicious cycle of pain and antalgic postures acquired over time. The mean duration of symptoms among women with CPP was about five years, and postural impairments over time can contribute significantly to the maintenance or worsening of pain [11,17-20]. Nevertheless, we cannot conclude that women with CPP always show the same postural pattern. First, although we observed an association between CPP and postural changes, the control group, consisting of women who did not report any type of pain, also presented with several postural changes. We believe that postural changes among controls occurred because posture depends not only on pathologic condition, but on several other factors, including habits acquired by individuals throughout life, their work activities, and even their emotional and psychological states. Second, our study design did not allow us to determine whether postural changes were the cause or consequence of CPP. However, identifying postural changes is an important part of evaluating women with CPP because improvements in posture can contribute to improvement in CPP symptoms.

In this study, posture was assessed in a strictly clinical manner, with the examiners recording the static posture adopted by the women. This method of assessment was used because we wanted to determine the efficacy and reproducibility of this type of evaluation so that it might be incorporated into clinical practice in the evaluation of women with CPP. Because of its simplicity, this type of examination can be easily performed during ambulatory patient care at any level of assistance, thus minimizing factors that may worsen or perpetuate CPP and helping to refer these women to specialized services. However we recognize that, scientifically, more objective forms of postural assessment such as biophotogrammetry are necessary. However we believe that the method described here may be useful in assessing the effects of physiotherapy and/or advice to alleviate pain in women with CPP who have musculoskeletal changes.

Our findings also support the importance of multidisciplinary care, involving physicians, physical therapists and psychologists, for women with CPP. In this series, musculoskeletal changes were associated with CPP in at least in 34% of the women in the CPP group, indicating that a more detailed assessment of women with CPP is necessary for better diagnosis and to provide more effective treatment for these women, including control of situations that may reduce the pain threshold.

## Conclusion

From this study we conclude that postural changes are seen more frequently in women with CPP. However, it is not possible to confirm if these changes are causes or consequences of CPP. Thus, more detailed assessments are necessary to obtain better differential diagnosis and, consequently, more effective treatment for these women.

## Competing interests

The authors declare that they have no competing interests.

## Authors' contributions

MLLSM, ELMV and OBPN contributed to concept and design of study. MLLSM, OBPN and FJCR contributed to analysis and interpretation and preparation of the manuscript. MLLSM, OBPL, JCRS and AAN contributed to patient recruitment, data collection and editing the final manuscript. All authors gave final approval to the article submitted.

## Pre-publication history

The pre-publication history for this paper can be accessed here:


